# Anderson's disease/chylomicron retention disease in a Japanese patient with uniparental disomy 7 and a normal *SAR1B *gene protein coding sequence

**DOI:** 10.1186/1750-1172-6-78

**Published:** 2011-11-21

**Authors:** Tomoo Okada, Michio Miyashita, Junji Fukuhara, Masahiko Sugitani, Takahiro Ueno, Marie-Elisabeth Samson-Bouma, Lawrence P Aggerbeck

**Affiliations:** 1 Department of Pediatrics, Nihon University School of Medicine, 30-1 Oyaguchi Kamicho Itabashi-ku, Tokyo 173-8630, Japan; 2 Divison of Pathology, Department of Pathology and Microbiology, Nihon University School of Medicine, 30-1 Oyaguchi Kamicho Itabashi-ku, Tokyo 173-8630, Japan; 3 Department of Nephrology, hypertension and endocrinology, Nihon University School of Medicine, 30-1 Oyaguchi Kamicho Itabashi-ku, Tokyo 173-8630, Japan; 4 INSERM U698, Hémostase, bio-ingénierie, immunopathologie et remodelage cardiovasculaires, Université Paris VII, D. Diderot, CHU X. Bichat, secteur Claude Bernard, 46 rue Henri Huchard, 75877 Paris cedex 18, France; 5 INSERM UMR-S 747, Toxicologie, Pharmacologie et Signalisation Cellulaire, Centre Universitaire des Saints-Pères, Université Paris Descartes, 45 rue des Saints-Pères, 75006 Paris, France

## Abstract

**Background:**

Anderson's Disease (AD)/Chylomicron Retention Disease (CMRD) is a rare hereditary hypocholesterolemic disorder characterized by a malabsorption syndrome with steatorrhea, failure to thrive and the absence of chylomicrons and apolipoprotein B48 post-prandially. All patients studied to date exhibit a mutation in the *SAR1B *gene, which codes for an essential component of the vesicular coat protein complex II (COPII) necessary for endoplasmic reticulum to Golgi transport. We describe here a patient with AD/CMRD, a normal *SAR1B *gene protein coding sequence and maternal uniparental disomy of chromosome 7 (matUPD7).

**Methods and Results:**

The patient, one of two siblings of a Japanese family, had diarrhea and steatorrhea beginning at five months of age. There was a white duodenal mucosa upon endoscopy. Light and electron microscopy showed that the intestinal villi were normal but that they had lipid laden enterocytes containing accumulations of lipid droplets in the cytoplasm and lipoprotein-size particles in membrane bound structures. Although there were decreased amounts in plasma of total- and low-density lipoprotein cholesterol, apolipoproteins AI and B and vitamin E levels, the triglycerides were normal, typical of AD/CMRD. The presence of low density lipoproteins and apolipoprotein B in the plasma, although in decreased amounts, ruled out abetalipoproteinemia. The parents were asymptomatic with normal plasma cholesterol levels suggesting a recessive disorder and ruling out familial hypobetalipoproteinemia. Sequencing of genomic DNA showed that the 8 exons of the *SAR1B *gene were normal. Whole genome SNP analysis and karyotyping revealed matUPD7 with a normal karyotype. In contrast to other cases of AD/CMRD which have shown catch-up growth following vitamin supplementation and a fat restricted diet, our patient exhibits continued growth delay and other aspects of the matUPD7 and Silver-Russell Syndrome phenotypes.

**Conclusions:**

This patient with AD/CMRD has a normal *SAR1B *gene protein coding sequence which suggests that factors other than the SAR1B protein may be crucial for chylomicron secretion. Further, this patient exhibits matUPD7 with regions of homozygosity which might be useful for elucidating the molecular basis of the defect(s) in this individual. The results provide novel insights into the relation between phenotype and genotype in these diseases and for the mechanisms of secretion in the intestine.

## Background

The differential diagnosis in a young child with diarrhea, vomiting and failure to thrive accompanied by hypocholesterolemia includes secondary hypocholesterolemias and the three familial hypocholesterolemias: Hypobetalipoproteinemia (HBL), Abetalipoproteinemia (ABL) and Anderson's Disease (AD)/Chylomicron Retention Disease (CMRD) [1]. Among the characteristics that distinguish these diseases are the complete absence of low density lipoproteins (LDL) along with very low levels of plasma triglycerides (TGs) in ABL and homozygous HBL and low (but not absent) levels of LDL and low levels of plasma TGs in heterozygous HBL. In contrast, low (but not absent) levels of plasma LDL and normal levels of plasma TGs in the patient along with normal plasma cholesterol values in the parents are indicative of AD/CMRD.

AD/CMRD (OMIM 246700) is a rare (less than 50 cases described) recessively inherited malabsorption syndrome with steatorrhea [1,2]. The small intestine, when visualized by endoscopy, exhibits a characteristic white coating. Microscopically, the intestine has normal villi but the enterocytes are fat-laden and contain large amounts of large lipid droplets free in the cytoplasm along with lipoprotein-sized structures in membrane bound compartments. In the blood, there is a selective absence of chylomicrons (and of apoB48) in the postprandial state and low levels of plasma total lipids, cholesterol, phospholipids, carotenoids, lipid soluble vitamins (particularly vitamin E), low density (LDL) and high density (HDL) lipoproteins, apoB, and apoAI.

Of about 50 patients that have been described in the literature as having AD/CMRD, 34 have mutations (15 different mutations, frameshifts, missense or deletions) in the *SAR1B (*formerly *SARA2*, OMIM 607690*) *gene [1] (see also additional file 1 of [2]). Information concerning the *SAR1B *genes in the other patients has not been published. The protein product of this gene, SAR1B, belongs to the Sar1-ADP ribosylation factor family of small GTPases and plays a critical role in the vesicular coat protein complex II (COPII)-dependent transport of proteins from the endoplasmic reticulum to the Golgi apparatus [3]. All the reported mutations in the *SAR1B *gene are predicted to lead to the incapacitation of SAR1B protein function in COPII-dependent transport and, thus, are expected to result in a defect in chylomicron secretion.

There are multiple components in the COPII-dependent transport mechanism and recent work has indicated that lipoprotein secretion may be more complex than previously thought and not involve the components of the COPII-dependent transport mechanism in a "classical" way [4,5]. Thus, it might be expected that mutations in genes other than *SAR1B *might lead to the AD/CMRD phenotype.

In this paper, we describe the clinical manifestations and biochemical characterization of a patient with AD/CMRD who does not have a mutation in the *SAR1B *gene protein coding sequence and who was found also to have maternal uniparental disomy of chromosome 7 (matUPD7).

## Patient and methods

### Patient

The patient, a boy born in 2005, was the first of 2 children (a sister was born in 2008) of unrelated Japanese parents. The pregnancy was complicated by hypertension and oligohydroamnia, and intra-uterine fetal growth retardation was noted. After 37 weeks and 5 days of gestation, birth was by uneventful vaginal delivery. The patient's birth weight, body length, head circumference and the chest circumference were all abnormally low (Table 1). Three days following birth, the patient had early onset jaundice with induced ABO isoimmunization which was successfully treated with phototherapy. At the age of 5 months, fatty, malodorous stools were noted. The weight gain was extremely poor despite the intake of 600 - 700 ml formula per day. Total parenteral nutrition, including essential fatty acids and fat soluble vitamins, was instituted.

**Table 1:** Clinical and biological data of the patient.

Parameter	Patient
Parental consanguinity	No
Age of onset of symptoms	5 months
*Growth retardation (at birth)*	
Weight (SDS)	-2.93
Height (SDS)	-3.71
Head circumference (SDS)	-2.02
Chest circumference (%tile)	< 10
Digestive symptoms	Diarrhea
Deep tendon reflexes	Present, normal
Hematological (Acanthocytes)	Absent
Steatorrhea (g/24 hr)	12
*Serum lipid (g/L) and vitamin values*	
Cholesterol (N: 1.2 - 2.2)	1.17, 1.09
LDL-cholesterol (N: 0.60 - 1.30)	0.57, 0.51
HDL-cholesterol (N > 0.40)	0.44, 0.43
Triglycerides (N: 0.27 - 1.20)	1.08, 1.17
ApoAI (N: 1.45+/-0.16)	1.19, 1.27
ApoB (N: 0.75+/-0.14)	0.52, 0.46
ALA (18:3n3) (N: 0.42-1.3 w/w%)	0.66
AA (20:4n6) (N: 4.19-9.57 w/w%)	3.8
EPA (N: 0.54-5.20 w/w%)	0.59
DHA (N: 2.33-7.34 w/w%)	2.32
Vitamin E (N: 7.5-14.1 mg/L)	6.6
Vitamin A (N: 970-3160 IU/L)	1060
AST (xULN) ALT (x ULN)	1.7 Normal
CK (x ULN)	Normal
Endoscopy: white hoary frosting	+++
Intestinal biopsy: fat laden enterocytes	+++
*Ultrastructural analysis*	
Lipid droplets	+++
Membrane bound lipoprotein-like particles	+++
Response to oral fat load	No chylomicrons

SDS: Standard deviation score, ALA: Alpha linolenic acid; AA: Arachidonic acid; EPA: Eicosapentaenoic acid; DHA: Docosahexaenoic acid;ULN: Upper limit of normal; +, ++, +++: present in increasing amounts; AST: Aspartate Amino Transferase; ALT: Alanine Amino Transferase; CK: Creatine kinase. The values were obtained at 6-11 months of age.

At 9 months of age, the patient was referred to the Department of Pediatrics of the Nihon University School of Medicine for failure to thrive and steatorrhea. Physical examination showed a body weight of 4192 g (-4.79 Standard Deviation Score, SDS) and a body length 59.5 cm (-4.3 SDS). The neurological and opthalmological examinations were normal.

Laboratory tests (Table 1 and Additional file 1: Additional blood chemistry values for the patient) showed decreased levels of plasma total- and LDL-cholesterol with low normal levels of HDL-cholesterol. In contrast, the plasma TG level was normal. The plasma apolipoprotein profile was also altered, with decreased levels of apoB, apoAI, apoAII and apoE. The levels of vitamin E, arachidonic acid (20:4 n6) and non-esterified fatty acids were also low. No acanthocytes were observed. The value for AST was slightly increased. The growth hormone level, after arginine stimulation, was considered to be low. After milk feeding, there was no increase in TG levels as compared to the fasting value and no chylomicrons were observed. Typically, in normolipidemic subjects, although a fat load produces no significant change in total cholesterol concentration, the triglycerides increase after a fat meal and chylomicrons are easily demonstrable [6].

The patient's father has no significant medical problems. The patient's mother is obese (body mass index = 32.3 kg/m^2^) and has a fatty liver with hypertriglyceridemia, but no other known abnormalities. Both parents have normal total-, LDL- and HDL-cholesterol levels (Additional file 2: Blood lipid and other chemistry values for the parents). Other biochemical parameters for the parents were normal except for, in the mother only, slightly increased, apoCII, Lp(a), AST and ALT.

Based upon the clinical and laboratory findings and current diagnostic criteria [1], a preliminary diagnosis of AD/CMRD was made for the patient and further studies were undertaken to confirm the diagnosis.

## Methods (see also Additional file 3: Supplemental methods)

Blood sampling, intestinal biopsies and genetic analyses were approved by the University Ethics Committee (Nihon University, Itabashi Hospital). Informed, written consent was obtained from the parents of the children and blood samples from the other family members were obtained following informed consent of the individual. The study was also performed in the context of a protocol approved by INSERM (RBM 0256) and by a bioethics committee (Comité Consultatif de Protection des Personnes dans la Recherche Biomédicale de Paris Bichat-Claude Bernard, Paris, France, CCPPRB Bichat-C. Bernard-2003/05).

At 19 months of age, jejunal biopsies were obtained two hours following fat loading (50 g olive oil per 1.73 m^2 ^surface area) via nasal-gastric tube into the stomach and were examined by light and electron microscopy according to standard procedures. Venous blood samples were drawn after a 12-hour fast. Blood lipid, lipoprotein and apolipoprotein analyses, polyacrylamide gel electrophoresis and ultracentrifugation were performed according previously described procedures. Genomic DNA was isolated from whole blood and the 8 exons of the *SAR1B *gene and their flanking intronic sequences were sequenced by standard methods. Whole genome SNP analysis and cytogenetic analysis was performed as described in Additional file 3: Supplemental methods.

## Results

### Intestinal morphology and ultrastructure

**Figure 1:**
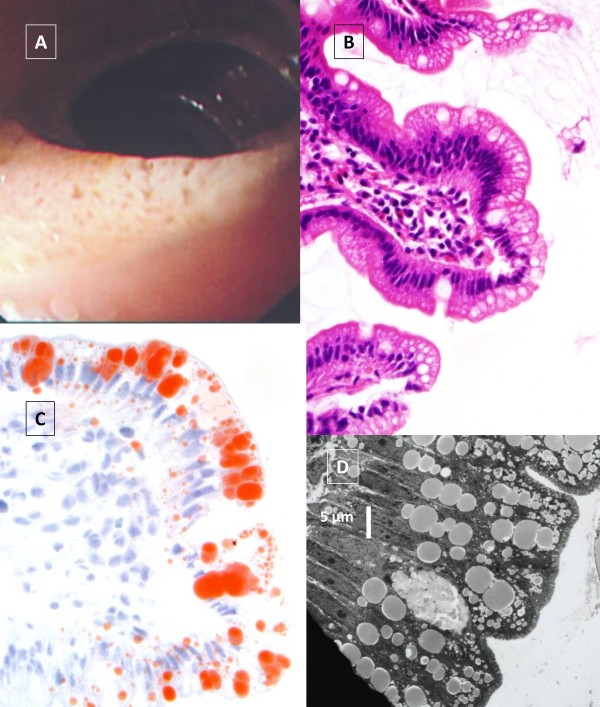
**Intestinal endoscopy, light and electron microscopy of the intestinal biopsy**. Video-endoscopy of the jejunum of patient (A) shows the typical white coating (« white hoary frosting ») on the small intestinal mucosa, which is not observed in a normal subject. Light microscopy of the jejunal biopsy stained with hematoxylin/eosin (B) shows vacuolated enterocytes that stain positively with Oil Red O predominantly at the tips of the *villi *(C). Goblet cells are normal. Electron microscopy at low magnification of the biopsy shows large lipid droplets in the cytoplasm as well as smaller lipoprotein-sized particles (D).

**Figure 2:**
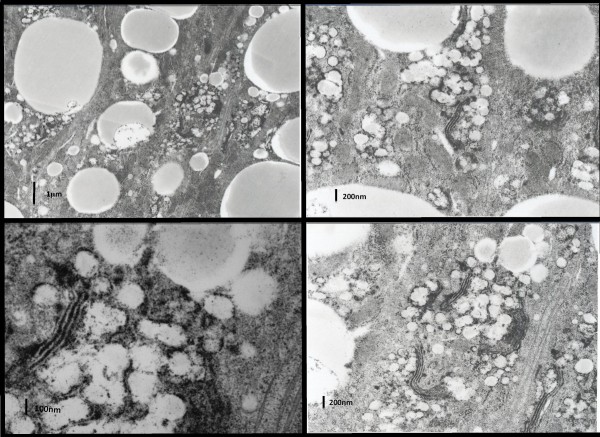
**Electron microscopy of the intestinal biopsy**. The enterocytes exhibit large lipid droplets in the cytoplasm. At higher magnification, numerous lipoprotein-sized particles are apparent which appear to be surrounded by a membrane near a Golgi apparatus which appears devoid of particles. The intercellular spaces are empty.

Intestinal video-endoscopy showed a white coating on the mucosa (Figure 1A). The intestinal biopsy exhibited normal *villi *(Figure 1B), however, the enterocytes were overloaded with fat droplets, that stain with Oil Red O, mainly at the tip of the *villus *(Figure 1C). Electron microscopic examination of the biopsies showed that the enterocytes contained an accumulation of numerous free lipid droplets (Figure 1D and Figure 2) as well as membrane bound lipoprotein-sized structures that accumulated in the cytoplasm of the enterocytes (Figure 2).

### SAR1B gene sequencing, whole genome SNP and karyotype analyses

**Figure 3:**
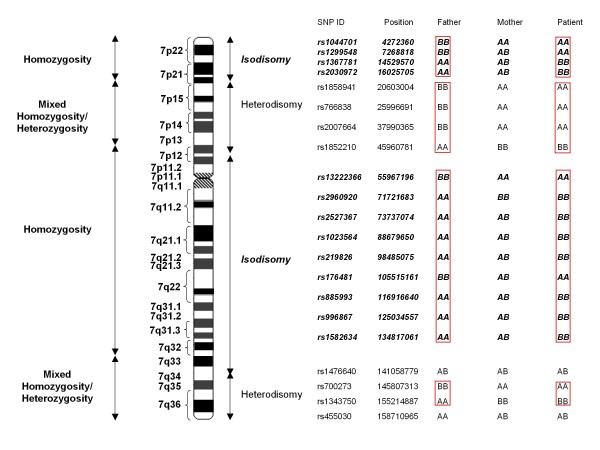
**Maternal uniparental disomy of chromosome 7 (matUPD7)**. An ideogram of chromosome 7 is shown surrounded, on the right, by a portion of the genotypes of the patient and his father and mother obtained by SNP analysis and the regions of isodisomy and heterodisomy that are inferred from the analysis. On the left, regions of complete homozygosity and regions containing both homozygosity and heterozygosity as determined by SNP analysis are shown. Red boxes show regions of non-Mendelian inheritance.

Sequencing of all 8 exons of the *SAR1B *gene, including the intron-exon boundaries revealed no mutations. Whole genome SNP analysis showed a loss of heterozygosity over extensive regions of chromosome 7 (Additional file 4: Loss of heterozygosity on chromosome 7). Maternal uniparental disomy (matUPD7) of the entire chromosome was found with regions of both isodisomy and heterodisomy (Figure 3 and Additional file 5: Genotypes and inferred regions of isodisomy and heterodisomy). There was a normal 46XY karyotype without any chromosomal abnormalities.

### Patient course

At 3 years of age, after having followed a diet restricted in fat accompanied by **v**itamin E and essential fatty acid supplementation and a 60% Medium Chain Triglycerides (MCT) formula, the diarrhea had improved and the steatorrhea ceased. The growth curve remained below the normal range for both weight and height (Additional file 6: Growth curves, growth track and head circumference of the patient). There was no micrognathia as judged by X-ray upon admission and dental development has not been delayed although there is a slight malocclusion and the patient has some difficulty swallowing. The patient has exhibited slow feeding since he was an infant and he sometimes sweats while asleep.

At 4 years and 10 months of age the patient remained small in stature and was severely underweight but the head circumference was within normal limits (Additional file 6: Growth curves, growth track and head circumference of the patient). His bone age as assessed by X-ray (3 years old) was delayed as compared to his chronological age (4.83 years). His IQ is 81(borderline). He has a slightly triangular face with a small chin and thin upper lip but without frontal bossing or low set ears. There is no clino or brachydactyly. No neurological deficits were found. There are no muscular symptoms (and in particular no muscular hypotonia). There are no neuro-ophtalmic or cardiac abnormalities. The kidneys were normal by ultrasound and computed tomography. There is no hypospadia or cryptorchidism. However, the patient had delayed speech with episodes of asthmatic bronchitis (up to 4 years old) but without any family history of asthma or allergy. Following vitamin E supplementation (α-tocopherol 50 mg/kg/d), the plasma vitamin E level became normal. The total plasma cholesterol, HDL-cholesterol and LDL-cholesterol values, however, remained low. The AST level continued to be slightly increased. Ultrasound revealed a fatty liver.

The results are characteristic of AD/CMRD. Several of the clinical features also recall a Silver-Russell Syndrome (SRS) or matUPD7 phenotype [7,8] whereas some other features of SRS or matUPD7 (camptodactyly, joint contractures, café au lait patches, limb asymmetry, muscular hypotonia, ataxia, dystonia, receptive language impairment, behavioral problems) were specifically evaluated and found to be absent.

## Discussion

Two distinct phenotypes are apparent in this patient; one consists primarily of the growth and developmental defects which are characteristic of cases of maternal uniparental disomy of chromosome 7 (matUPD7) and the other is composed of a lipid malabsorption disorder typical of Anderson's Disease/Chylomicron retention disease (AD/CMRD). The mixture of heterodisomy and isodisomy with isodisomy in the pericentric region suggests trisomy rescue or gamete complementation and a meiosis II error as a mechanism for the matUPD7 [9]. There does not appear to be a change in copy number. A trend of advanced mean maternal age in maternal heterodisomy but not isodisomy has been reported but the ages of the mother (28 years) and the father (31 years) at the birth of our patient were not particularly advanced [10,11].

Many of the clinical manifestations that the patient exhibits and which are found in other cases of what has been designated as the matUPD7 phenotype are found also in Silver-Russell Syndrome (SRS): the patient was small for gestational age (SGA) and exhibited intrauterine (IUGR) and post-natal growth retardation (PNGR), failure to thrive, speech difficulties, relative macrocephaly, and a somewhat triangular face [7,8,12,13]. MatUPD7 is reported to occur in about 5-10% of patients diagnosed as having SRS, which has been described as a clinically and genetically heterogeneous disorder [8]. Other frequently observed features of SRS are craniofacial anomalies (a small chin with a thin upper lip, micrognathia, downturned corners of the mouth and ear anomalies), clinodactyly of the 5^th ^finger and limb and body asymmetry (present in more than 50% of the patients). Several other features are less constant. The diagnosis of SRS is reported to depend upon the clinical expertise of the physician and the criteria for inclusion [8,12,14], however, using the scale proposed by Bartholdi and colleagues [14], our patient probably would not be classified as having SRS.

The expressive language impairment that our patient exhibits resembles the developmental delays (developmental verbal dyspraxia, DVD) that have been found to be associated with mutations in the *FOXP2 *gene (Forkhead-box P2) which is located on chromosome 7 [15]. This gene is located in area of isodisomy, in our patient, in which both copies of the gene are of maternal origin. DVD is not a frequent feature in SRS patients but it is more prevalent in a subgroup of patients with matUPD7. The dyspraxia phenotype in matUPD7 resembles that found in individuals with *FOXP2 *deletions but is milder. It has been suggested that the *FOXP2 *locus is differentially regulated in a parent-of-origin specific fashion [15]. The speech and language phenotype of patients with matUPD7 may be related to the absence of paternal *FOXP2*. A further explanation could be an effect on expression of an imprinted gene on chromosome 7 involved in the regulation of FOXP2 expression (or one of its isoforms).

The small gestational age (SGA) and intrauterine growth restriction (IUGR) in our patient could be related to the pre-eclampsia and oligohydroamnia as well as to the chromosome abnormality. The level of growth hormone, following arginine stimulation was considered to be low. A low level of growth hormone was reported in the first patient described with matUPD7 and appears to more common in cases of maternal heterodisomy as opposed to maternal isodisomy of chromosome 7 [7]. The growth delay present prenatally in maternal UPD7 has been reported to continue to adulthood [16]. In this respect, it is noteworthy that, in contrast to other patients with AD/CMRD, our patient has not shown signs of catch up growth following dietary modification and vitamin supplementation and the height and weight standard deviation score (SDS) curves remain flat (Additional file 6: Growth curves, growth track and head circumference of the patient).

It is not clear whether the defect in chylomicron secretion (AD/CMRD phenotype) arises due to mutation(s) associated with recessive disease present on chromosome 7 in the heterozygous state in the mother which become(s) homozygous in our patient due to the matUPD7 or whether both parents are heterozygous for the recessive disease mutation on the same or another chromosome and which has become homozygous in the patient. This is, however, the first patient with matUPD7 who exhibits a malabsorption phenotype. Each case of matUPD7 is unique and we conclude that the chromosomal regions that are altered in the other matUPD7 patients do not affect any of the genes that are involved in lipoprotein assembly and secretion. Nevertheless, in our patient, since matUPD7 leads to regions of chromosome 7 which are homozygous for one allele deriving from the mother which are different from those in other individuals with matUPD7, there is the possibility for this being the mechanism leading to the AD/CMRD phenotype.

All the other patients with AD/CMRD, for which the gene defect has been established, exhibit mutations in the *SAR1B *gene (located on chromosome 5) which are believed to incapacitate the SAR1B protein in its role in COPII-dependent transport and result in the defect of secretion of chylomicrons in the intestine. This is first description of a patient with AD/CMRD without a mutation in the protein coding region of the *SAR1B *gene. Although our studies do not address the possibility of a mutation deep within an intron or in the 5' or 3' ends of the gene, the relative frequencies of types of mutations underlying disease phenotypes (data from the Human Gene Mutation Database, 2011) show that the majority of mutations in mendelian disorders are missense or nonsense mutations, affect splice acceptor and donor sites or correspond to insertions, deletions and duplications which disrupt coding sequences. Non-coding or regulatory sequence variants are much less common and appear to be more likely to have neutral or weak effects or phenotypes, even in well-conserved non-coding sequences [17-19]. Nevertheless, local or distant non-coding genomic elements (promoters, enhancers, repressors) can play important roles in regulating gene expression [20] and we cannot rule out a mutation of this type which might affect, via transcriptional control, the expression of the SAR1B protein in this patient.

Phenotypes similar to AD/CMRD could arise from mutations in components other than SAR1B. The tissue-specific pathology in AD/CMRD patients could arise from mutations in components of paralogue- and cargo-specific mechanisms of transport analogous to other diseases associated with secretory pathway components (cranio-lenticulo-sutural dysplasia CLSD [21] and congenital dyserythropoietic anemia type II, CDAII, [22] in humans and craniorachischisis in mice [23]).

The intestine-specific manifestations in AD/CMRD might also arise from trafficking pathways for lipoprotein secretion that are different in the liver and the intestine. An intestinal chylomicron-specific transport mechanism that employs a pre-chylomicron transport vesicle (PCTV) has been proposed as an alternative to the "classical" COPII secretory pathway [4,5] and a VLDL-specific transport vesicle, the VTV, has been proposed to function in the liver [24,25]. These transport mechanisms use some elements of the "classical" COPII coat as well as other protein constituents. This could explain the few hepatic manifestations in most cases of AD/CMRD. Futher, additional, specific proteins may control the loading of very large cargoes by regulating the size of COPII carriers as, for example TANGO1 and cTAGE5 in the case of Collagen VII secretion [3,26].

In conclusion, our patient is not unique. In addition to the family described here, we have now found three other families having a total of 5 additional affected individuals that exhibit the AD/CMRD phenotype (but without matUPD7) for which the protein coding sequence of the *SAR1B *gene is normal, as in this patient. We are currently in the process of defining the genetic defect(s) in these families.

## Conclusions

This is the first report of the AD/CMRD and matUPD7 phenotypes in the same individual. The absence of a mutation in the *SAR1B *gene protein coding sequence suggests that a factor (or factors) other than the SAR1B protein may play(s) an essential role in chylomicron secretion. The presence of matUPD7 and the regions of homozygosity on chromosome 7 might be regions in which to search for these factors. The results shed new light on the mechanisms involved in intracellular transport and in the secretion of soluble cargos.

## Consent

Informed consent was obtained from each individual and from the patient's parents for the participation in and the publication of this study.

## Competing interests

LPA and MES-B have received grant support from Roche.

## Authors' contributions

TO, MM and JF provided clinical care for the patients and participated in the generation and analysis of the clinical data. TO and MM contributed to the clinical follow-up of the patient. MS arranged intestinal pathological analyses. TU analyzed SNP and isolated genomic DNA from whole blood. LPA coordinated and analyzed the whole genome SNP analysis. MES-B helped analyze the data. Each author read the text and participated in the writing of the manuscript. All authors read and approved the final manuscript.

## Supplementary Material

Additional file 1 **Additional blood chemistry values for the patient**. Click here for file

Additional file 2 **Blood lipid and other chemistry values for the parents**. Click here for file

Additional file 3 **Supplemental methods**. Click here for file

Additional file 4 **Loss of heterozygosity on chromosome 7**. Extensive regions in which there is a loss of heterozygosity as assessed by SNP analysis are shown on a diagram of chromosome 7. Large regions around p22, p21 p12 and q11.2, q21, q22, q31 and q32 have no B-allele frequencies equal to 0.5. Click here for file

Additional file 5 **Genotypes and inferred regions of isodisomy and heterodisomy**. This file provides the SNP determined genotypes for the patient, his parents and his sister and regions of isodisomy (green) and heterodisomy (blue) inferred from the results. In yellow, the genotype of the sister is identical to the genotype of the brother. Regions in red indicate non-Mendalian inheritance in the patient as compared to his father. Click here for file

Additional file 6 **Growth curves, growth track and head circumference of the patient**. The patient has below normal height and weight and does not show catch up growth. The head circumference of the patient, which was initially small, became essentially normal, which when combined with the low weight and size is consistent with a relative macrocephaly. The standard deviation scores (SDS) for the patient's height, weight and head circumference are plotted as a function of age. There is marked below normal height and weight with a relatively normal head circumference. Click here for file

## References

[B1] Peretti N Sassolas A Roy CC Deslandres C Charcosset M Castagnetti J Pugnet-Chardon L Moulin P Labarge S Bouthillier L Lachaux A Levy E Guidelines for the diagnosis and management of chylomicron retention disease based on a review of the literature and the experience of two centers Orphanet Journal of Rare Diseases 2010 5 24 37 10.1186/1750-1172-5-24 20920215 PMC2956717

[B2] Georges A Bonneau J Bonnefont-Rousselot D Champigneulle J Rabès JP Abifadel M Aparicio T Guenedet JC Bruckert E Boileau C Morali A Varret M Aggerbeck LP Samson-Bouma ME Molecular analysis and intestinal expression of *SAR1 *genes and proteins in Anderson's disease (Chylomicron retention disease) Orphanet Journal of Rare Diseases 2011 6 1 10.1186/1750-1172-6-1 21235735 PMC3029219

[B3] Jensen D Schekman R COPII-mediated vesicle formation at a glance J Cell Sci 2011 124 1 4 21172817 10.1242/jcs.069773

[B4] Mansbach CM Siddiqi SA The biogenesis of chylomicrons Annu Rev Physiol 2010 72 315 333 20148678 10.1146/annurev-physiol-021909-135801 PMC4861230

[B5] Siddiqi S Saleem U Abumrad N Davidson N Storch J Siddiqi SA Mansbach CM A Novel Multi-protein Complex Is Required for the Generation of the Pre-chylomicron Transport Vesicle from Intestinal ER J Lipid Res 2010 51 1918 1928 10.1194/jlr.M005611 20237389 PMC2882727

[B6] Patsch JR Miesenbock G Hopferwieser T Muhlberger V Knapp E Dunn JK Gotto AM Patsch W Relation of triglyceride metabolism and coronary artery disease. Studies in the postprandial state Arterioscler Thromb 1992 12 1336 1345 10.1161/01.ATV.12.11.1336 1420093

[B7] Kotzot D Maternal uniparental disomy 7 and Silver-Russell syndrome-Clinical update and comparison with other subgroups Eur J Med Genet 2008 51 444 451 10.1016/j.ejmg.2008.06.001 18655849

[B8] Eggermann T Russell-Silver syndrome Am J Med Genet C Semin Med Genet 2010 154C 355 364 10.1002/ajmg.c.30274 20803658

[B9] Yamazawa K Ogata T Ferguson-Smith AC Uniparental disomy and human disease: an overview Am J Med Genet C Semin Med Genet 2010 154C 329 34 10.1002/ajmg.c.30270 20803655

[B10] Kotzot D Advanced parental age in maternal uniparental disomy (UPD): implications for the mechanism of formation Eur J Hum Genet 2004 12 343 346 10.1038/sj.ejhg.5201158 14747835

[B11] Wakeling EL Amero SA Alders M Bliek J Forsythe E Kuman S Lim DH MacDonald F Mackay DJ Maher ER Moore GE Poole RL Price SM Tangeraas T Turner CLS Van Haelst MM Willoughby C Temple IK Cobben JM Epigenotype-phenotype correlations in Silver-Russell syndrome J Med Genet 2010 47 760 768 10.1136/jmg.2010.079111 20685669 PMC2976034

[B12] Price SM Stanhope R Garrett C Preece MA Trembath RC The spectrum of Silver-Russell syndrome: a clinical and molecular genetic study and new diagnostic criteria J Med Genet 1999 36 837 842 10544228 PMC1734267

[B13] Kotzot D Complex and segmental uniparental disomy updated J Med Genet 2008 45 545 556 10.1136/jmg.2008.058016 18524837

[B14] Bartholdi D Krajewska-Walasek M Ounap K Gaspar H Chrzanowska KH Ilyana H Kayserili H Lurie IW Schinzel A Baumer A Epigenetic mutations of the imprinted IGF2-H19 domain in Silver-Russell syndrome (SRS): Results from a large cohort of patients with SRS and SRS-like phenotypes J Med Genet 2009 46 192 197 19066168 10.1136/jmg.2008.061820

[B15] Feuk L Kalervo A Lipsanen-Nyman M Skaug J Nakabayashi K Finucane B Hartung D Innes M Kerem B Nowaczyk MJ Rivlin J Roberts W Senman L Summers A Szatmari P Wong V Vincent JB Zeesman S Osborne LR Cardy JO Kere J Sherer SW Hannula-Jouppi K Absence of a paternally inherited FOXP2 gene in developmental verbal dyspraxia Am J Hum Genet 2006 79 965 972 10.1086/508902 17033973 PMC1698557

[B16] Kotzot D Growth parameters in maternal uniparental disomy Eur J Pediatr 2007 166 1143 1149 10.1007/s00431-006-0396-5 17203278

[B17] Botstein D Risch N Discovering genotypes underlying human phenotypes: past successes for mendelian disease, future approaches for complex disease Nat Genet Suppl 2003 33 228 237 10.1038/ng1090 12610532

[B18] Chen CTL Wang JC Cohen BA The strength of selection on ultraconserved elements in the human genome Am J Hum Genet 2007 80 692 704 10.1086/513149 17357075 PMC1852725

[B19] Ahituv N Zhu Y Visel A Holt A Afzal V Pennacchio LA Rubin EM Deletion of ultraconserved elements yields viable mice PLoS Biology 2007 5 1906 1911 10.1371/journal.pbio.0050234 PMC1964772 17803355

[B20] Kleinjan DA van Heyningen V Long-range control of gene expression: Emerging mechanisms and disruption in disease Am J Hum Genet 2005 76 8 32 10.1086/426833 15549674 PMC1196435

[B21] Fromme JC Ravazzola M Hamamoto S Al-Balwi M Eyaid W Boyadjiev SA Cosson P Schekman R Orci L The genetic basis of a craniofacial disease provides insight into COPII coat assembly Dev Cell 2007 13 623 634 10.1016/j.devcel.2007.10.005 17981132 PMC2262049

[B22] Schwarz K Iolascon A Verissimo F Trede NS Horsley W Chen W Paw BH Hopfner KP Holzmann K Russo R Esposito MR Spano D De Falco L Heinrich K Joggerst B Rojewski MT Perrotta S Denecke J Pannicke U Delaunay J Pepperkok R Heimpel H Mutations affecting the secretory COPII coat component SEC23B cause congenital dyserythropoietic anemia type II Nat Genet 2009 41 936 940 10.1038/ng.405 19561605

[B23] Merte J Jensen D Wright K Sarsfield S Wang Y Schekman R Ginty DD Sec24b selectively sorts Vangl2 to regulate planar cell polarity during neural tube closure Nat Cell Biol 2010 12 41 46 10.1038/ncb2002 19966784 PMC2823131

[B24] Siddiqi SA VLDL exits from the endoplasmic reticulum in a specialized vesicle, the VLDL transport vesicle, in rat primary hepatocytes Biochem J 2008 413 333 342 10.1042/BJ20071469 18397176

[B25] Siddiqi S Mani AM Siddiqi SA The Identification of the SNARE-complex Required for the Fusion of VLDL Transport Vesicle with Hepatic cis-Golgi Biochem J 2010 429 391 401 10.1042/BJ20100336 20450495 PMC4861233

[B26] Malhotra V Erlmann P Protein export at the ER: loading big collagens into COPII carriers The EMBO J 2011 30 3475 3480 10.1038/emboj.2011.255 PMC3181478 21878990

