# Supplementary material for: Anderson's disease/chylomicron retention disease in a Japanese patient with uniparental disomy 7 and a normal SAR1B gene protein coding sequence
Source: Orphanet J Rare Dis. 2011 Nov 21;6:78. doi: 10.1186/1750-1172-6-78 (PMC3284428; doi:10.1186/1750-1172-6-78)
Supplement: Additional file 1 — Additional blood chemistry values for the patient. [file 1750-1172-6-78-S1.PDF]

Additional file 1: Additional blood chemistry values for the patient.

Parameter	Patient
ApoAII (N: 0.31+/-0.047 g/L)	0.257, 0.277
ApoCII (N: 0.029+/-0.008 g/L)	0.032, 0.029
ApoCIII (N: 0.067+/-0.019 g/L)	0.065, 0.060
ApoE (N: 0.04+/-0.009 g/L)	0.031, 0.029
Lp(a) (N: <0.36 g/L)	0.10
NEFA (N: 140-850 μEq/L)	40
GH(N: >5 μg/L)	4.03
TSH (N: 0.6-6.3 mU/L)	5.59
FT3 (N: 2.29-4.58 ng/L)	3.53
FT4 (N: 11.4+/-2 ng/L)	16.6
ACTH (N: 18.7+/-6.8 ng/L)	9.3
AST (N: 8-38 IU/L)	65
ALT (N: 4-44 U/L)	29

GH: Growth hormone; NEFA: Non-esterified fatty acids. Values were obtained at 6-10 months of age.
