# Supplementary material for: Anderson's disease/chylomicron retention disease in a Japanese patient with uniparental disomy 7 and a normal SAR1B gene protein coding sequence
Source: Orphanet J Rare Dis. 2011 Nov 21;6:78. doi: 10.1186/1750-1172-6-78 (PMC3284428; doi:10.1186/1750-1172-6-78)
Supplement: Additional file 2 — Blood lipid and other chemistry values for the parents. [file 1750-1172-6-78-S2.PDF]

Additional file 2: Blood chemistry values of the parents

Parameter	Mother	Father
TC (N: <2.20 g/L)	2.01	1.90
HDL-C (N: >0.40 g/L)	0.47	0.48
LDL-C (N: <1.40 g/L)	1.14	1.19
TG (N: <1.50 g/L)	2.02	1.16
NEFA (N: 130-160 uEq/L)	290	400
Apo AI (N: >1.20 g/L)	1.48	1.39
Apo AII (N: >0.246 g/L)	0.262	0.263
Apo B (N: 0.66-1.09 g/L)	0.99	0.91
Apo CII (N: F<0.038; M<0.046 g/L)	0.040	0.044
Apo E (N: F<0.046; M<0.043 g/L)	0.041	0.038
Lp(a) (N: <0.36 g/L)	0.45	0.04
HDL (N: 20-55%)	34.3	32.3
LDL (N: 33-60%)	29.7	47.1
VLDL (N: 10-25%	20.2	20.6
AST (N: 8-38 IU/L)	45	20
ALT (N: 4-44 U/L)	51	28
