# Supplementary material for: Anderson's disease/chylomicron retention disease in a Japanese patient with uniparental disomy 7 and a normal SAR1B gene protein coding sequence
Source: Orphanet J Rare Dis. 2011 Nov 21;6:78. doi: 10.1186/1750-1172-6-78 (PMC3284428; doi:10.1186/1750-1172-6-78)
Supplement: Additional file 3 — Supplemental methods. [file 1750-1172-6-78-S3.PDF]

Additional file 3: Supplemental methods

Intestinal biopsies

Jejunal biopsies were performed two hours following fat loading (50g olive oil per 1.73m^2 surface area) via nasal-gastric tube into the stomach. Jejunal biopsy specimens were fixed in buffered formalin and further embedded in paraffin for standard histology or were directly frozen in liquid nitrogen for oil red O staining of the lipids. Specimens for electron microscopy were fixed in 2.5% glutaraldehyde for 4 hours and 1% osmium for 2 hours, dehydrated in graded ethanols, and embedded in epoxy resin. Ultrathin sections were cut on a Leica Ultracut UTC microtome (Leica Microsystems, Wetzlar, Germany), stained doubly with uranyl acetate and lead citrate, and observed in a JEOL JEM1200EX (Japan Electron Optics Laboratory, Tokyo, Japan) electron microscope.

Lipid and lipoprotein analysis

Venous blood samples were drawn after a 12-hour fast. Total cholesterol and TG concentrations were determined by automated enzymatic methods [1,2]. HDL-cholesterol–was determined by a direct method according to Egloff et al. [3]. LDL-cholesterol was calculated using Friedewald's equation [4]. ApoAI, apoAII, apoB, apoCII, apoCIII and apoE levels were measured by turbidimetric immunoassay using the corresponding antisera (Daiichi Pure Chemicals, Tokyo, Japan) [5] and taking into account the effects of age, sex and smoking. Serum lipoprotein analyses were performed by high performance liquid chromatography with gel permeation columns (LipoSEARCH; Skylight-Biotec, Inc., Akita, Japan) [6]. Cholesterol and TG levels were measured and SDS-polyacrylamide gel electrophoresis performed on each lipoprotein fraction. Agarose electrophoresis and ultracentrifugal isolation of lipoproteins were performed using standard procedures.

Gene sequencing of SAR1B, SNP and cytogenetic analyses

Genomic DNA was isolated from whole blood containing EDTA with a PSS Magtration 8Lx machine using a magnetic marble technique, according to the manufacturer's instructions. The 8 exons of the *SAR1B* gene and their flanking intronic sequences, were amplified by the polymerase chain reaction using the following primers:

Ex1-155F GAGACCCAGGCGGTGTTAGGGGTTG 25mer

Ex1-726R AGACCCGCCACCTTCACCTTACCAGG 26mer

Ex2-71F GACAGCTGAAGAGGCTGTGACACGG 25mer
Ex2-471R AGAGACCATCCTGGCTAACACGGTG 25mer
Ex3-194F GCTTCCCAAAGTGTGTAACTCTTTGTAG 28mer
Ex3-654R GGTAGGAATTATTACTGCCATTTTACAGAT 30mer
Ex4-175F CGGCCATGAATAGATTTAAAATAGCTTT 28mer
Ex4-589R CAGGAGACAGAGGTTGCAGTGAACT 25mer
Ex5-152F TGGCAGATATTTAAGAATGTTAGCACG 27mer
Ex5-573R ACATGCCTGTAATCCCAGCTATTGG 25mer
Ex6-170F ATATATTGTGAAGTACCAGCAGCTAAGG 28mer
Ex6-570R CTCCCTCTGCATTCTAAGTAATCACTAC 28mer
Ex7-263F AAGAACGTTGGTATGACATGATATGG 26mer
Ex7-663R TGAGGACATTAAAAAGACCCACAGG 25mer
Ex8-184F CCTGCCCTAAGGTAGAGTGAGAGAAG 26mer
Ex8-1167R GCCTTGAGAGCCTTAAACGCTATTC 25mer

Bidirectional fluorescent sequencing of purified PCR products (ExoSAP-IT, GE Healthcare) was carried out with the Big Dye® Terminator v1.1 Cycle Sequencing kit on an ABI-PRISM® 3100 genetic analyzer (Applied Biosystems). Electrophoretograms were analyzed using Gensearch DNA sequence analysis software (Phenosystems SA., Belgium).

Whole genome SNP analysis (6092 SNPs) was performed using the Illumina Infinium II Assay using a single bead type and dual color channel approach. BeadChips were imaged using the Illumina BeadArray Reader using BeadScan and BeadStudio software. The Illumina GenomeStudio Genotyping Module, QuantiSNP [7] and Homozygosity mapper [8] were used to analyze the data.

Cytogenetic analysis by Giesma staining of cultured peripheral blood leukocytes was performed with standard techniques [9].

1. Allain CC, Poon LS, Chan CS, Richmond W, Fu PC: **Enzymatic determination of total serum cholesterol**. *Clin Chem* 1974, **20**:470-475.
2. Fossati P, Prencipe L: **Serum triglycerides determined colorimetrically with an enzyme that produces hydrogen peroxide**. *Clin Chem* 1982, **28**:2077-2080.

3. Egloff M, Leglise D, Duvillard L, Steinmetz J, Boyer MJ, Ruelland A, Agher R, Beucler I: **[Multicenter evaluation on different analyzers of three methods for direct HDL-cholesterol assay]**. *Ann Biol Clin (Paris)* 1999, **57**:561-572.
4. Friedewald WT, Levy RI, Fredrickson DS: **Estimation of the concentration of low-density lipoprotein cholesterol in plasma, without use of the preparative ultracentrifuge**. *Clin Chem* 1972, **18**:499-502.
5. Sakurabayashi I, Saito Y, Kita T, Matsuzawa Y, Goto Y: **Reference intervals for serum apolipoproteins A-I, A-II, B, C-II, C-III and E in healthy Japanese determined with a commercial immunoturbidimetric assay and effects of sex, age, smoking, drinking, and Lp(a) level**. *Clin Chim Acta* 2001, **12**:87–95.
6. Okazaki M, Usui S, Ishigami M, Sakai N, Nakamura T, Matsuzawa Y, Yamashita S: **Identification of unique lipoprotein subclasses for visceral obesity by component analysis of cholesterol profile in high-performance liquid chromatography.** *Arterioscler Thromb Vasc Biol* 2005, **25**:578–84.
7. Colella S, Yau C, Taylor JM, Mirza G, Butler H, Clouston P, Bassett AS, Seller A, Holmes CC, Ragoussis J: **QuantiSNP: an Objective Bayes Hidden-Markov Model to detect and accurately map copy number variation using SNP genotyping data.** *Nucleic Acids Res* 2007, **35**:2013-25.
8. Seelow D, Schuelke M, Hildebrandt F, Nürnberg P: **Homozygosity Mapper—an interactive approach to homozygosity mapping.** *Nucleic Acids Research* 2009, **37**: W593–W599.
9. Giemsa G: **Eine Vereinfachung und Vervollkommnung meiner Methylenblau-Eosin-Färbemethode zur Erzielung der Romanowsky-Nocht'schen Chromatinfärbung.** *Centralblatt für Bakteriologie I Abteilung* 1904, **32**:307–313.
