# Supplementary figures and images for: Anderson's disease/chylomicron retention disease in a Japanese patient with uniparental disomy 7 and a normal SAR1B gene protein coding sequence
Source: Orphanet J Rare Dis. 2011 Nov 21;6:78. doi: 10.1186/1750-1172-6-78 (PMC3284428; doi:10.1186/1750-1172-6-78)

## Additional file 4: Loss of heterozygosity on chromosome 7

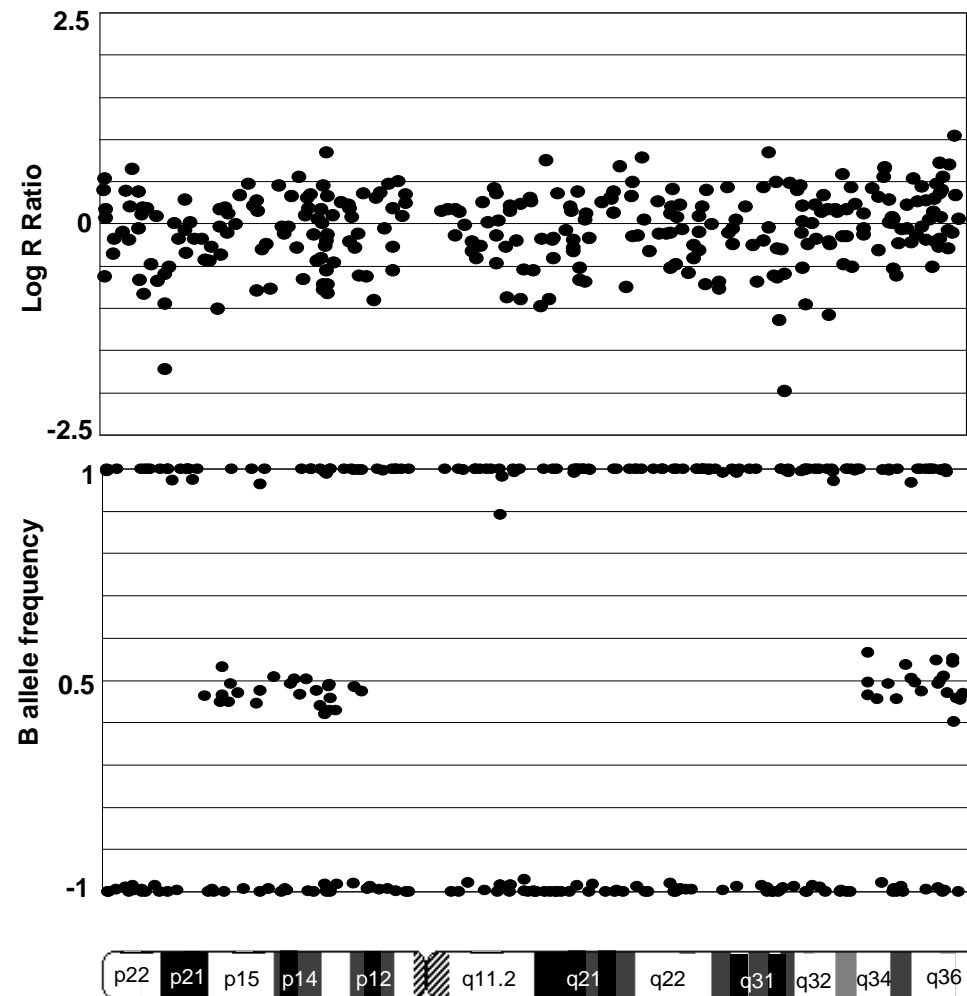

Supplement: Additional file 4 — Loss of heterozygosity on chromosome 7. Extensive regions in which there is a loss of heterozygosity as assessed by SNP analysis are shown on a diagram of chromosome 7. Large regions around p22, p21 p12 and q11.2, q21, q22, q31 and q32 have no B-allele frequencies equal to 0.5. [file 1750-1172-6-78-S4.PDF]

Additional file 6: Growth curves, growth track and head circumference of the patient

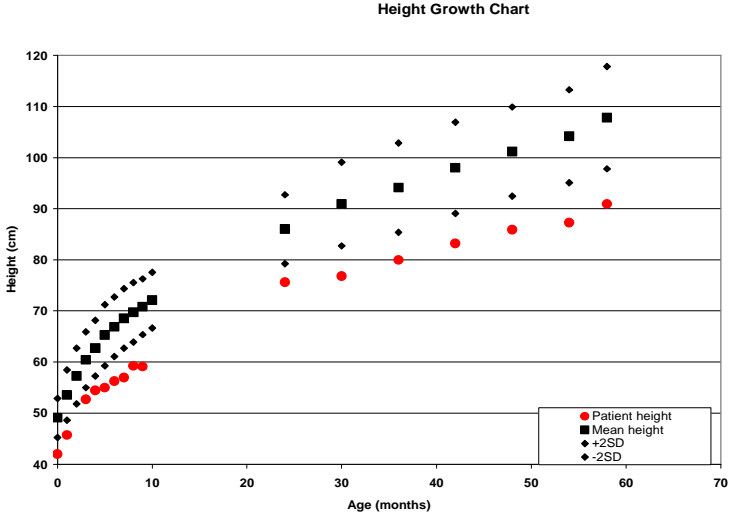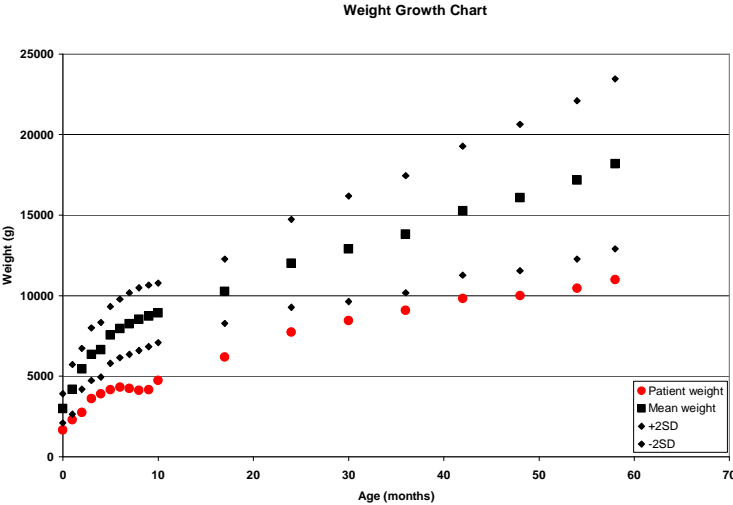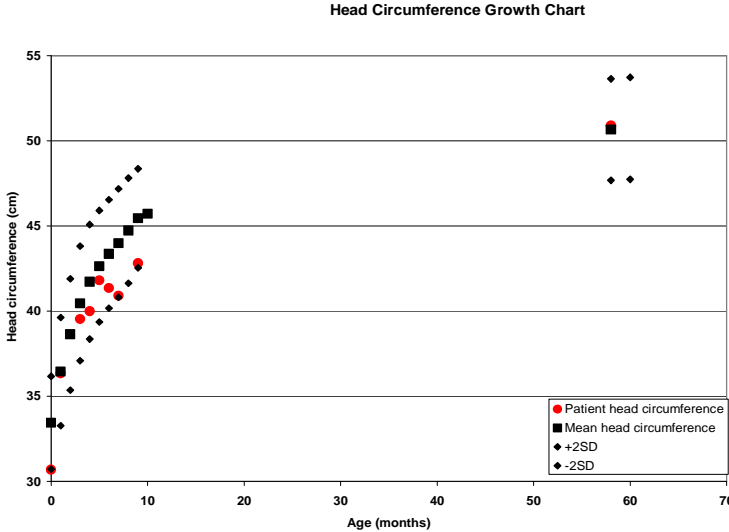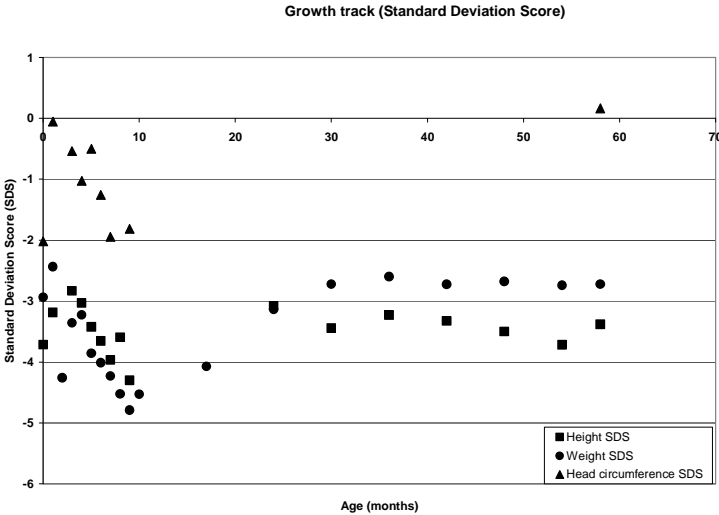

Supplement: Additional file 6 — Growth curves, growth track and head circumference of the patient. The patient has below normal height and weight and does not show catch up growth. The head circumference of the patient, which was initially small, became essentially normal, which when combined with the low weight and size is consistent with a relative macrocephaly. The standard deviation scores (SDS) for the patient's height, weight and head circumference are plotted as a function of age. There is marked below normal height and weight with a relatively normal head circumference. [file 1750-1172-6-78-S6.PDF]
