# Supplementary material for: Anderson's disease/chylomicron retention disease in a Japanese patient with uniparental disomy 7 and a normal SAR1B gene protein coding sequence
Source: Orphanet J Rare Dis. 2011 Nov 21;6:78. doi: 10.1186/1750-1172-6-78 (PMC3284428; doi:10.1186/1750-1172-6-78)
Supplement: Additional file 5 — Genotypes and inferred regions of isodisomy and heterodisomy. This file provides the SNP determined genotypes for the patient, his parents and his sister and regions of isodisomy (green) and heterodisomy (blue) inferred from the results. In yellow, the genotype of the sister is identical to the genotype of the brother. Regions in red indicate non-Mendalian inheritance in the patient as compared to his father. [file 1750-1172-6-78-S5.PDF]

Additional file 5: Genotypes and inferred regions of isodisomy and heterodisomy

SNP ID	Position	Genotype Father	Genotype Mother	Genotype Sister	Genotype Patient	Homozygosity or Heterozygosity (Patient)	Isodisomy or Heterodisomy (Patient)
rs1127460	845293	BB	BB	BB	BB	Homozyg.	Probable Isodisomy
rs7785205	891558	AB	BB	AB	BB	Homozyg.	Probable Isodisomy
rs1057558	1028892	AB	AA	AA	AA	Homozyg.	Probable Isodisomy
rs4236375	1111053	AB	AB	BB	BB	Homozyg.	Probable Isodisomy
rs1362136	2472093	AA	AA	AA	AA	Homozyg.	Probable Isodisomy
rs798485	2770036	AB	BB	BB	BB	Homozyg.	Probable Isodisomy
rs1044701	4272360	BB	AA	AB	AA	Homozyg.	Probable Isodisomy
rs1553960	4866621	AB	AA	AB	AA	Homozyg.	Probable Isodisomy
rs852432	5529266	AB	AA	AB	AA	Homozyg.	Probable Isodisomy
rs1468996	5700983	AB	AB	AB	AA	Homozyg.	Probable Isodisomy
rs4560	6029809	AB	AA	AA	AA	Homozyg.	Probable Isodisomy
rs13438509	7142832	BB	BB	BB	BB	Homozyg.	Probable Isodisomy
rs1299548	7268818	BB	AB	AB	AA	Homozyg.	matIsodisomy
rs1882600	7328048	AA	AA	AA	AA	Homozyg.	Probable Isodisomy
rs2042545	7841970	AB	BB	AB	BB	Homozyg.	Probable Isodisomy
rs37995	7995659	AA	AB	AA	AA	Homozyg.	Probable Isodisomy
rs1558557	8275518	AB	AB	AB	BB	Homozyg.	Probable Isodisomy
rs6463843	8805242	BB	BB	BB	BB	Homozyg.	Probable Isodisomy
rs1032873	9618805	AA	AA	AA	AA	Homozyg.	Probable Isodisomy
rs1013719	10521275	AA	AA	AA	AA	Homozyg.	Probable Isodisomy
rs1012123	10652456	AA	AB	AA	BB	Homozyg.	matIsodisomy
rs12666416	12008942	AB	BB	BB	BB	Homozyg.	Probable Isodisomy
rs2908740	12025775	AB	AB	BB	AA	Homozyg.	Probable Isodisomy
rs769111	12026331	AB	AB	AA	BB	Homozyg.	Probable Isodisomy
rs6460985	12890081	BB	AB	AB	BB	Homozyg.	Probable Isodisomy
rs2051930	13802528	AA	AA	AA	AA	Homozyg.	Probable Isodisomy
rs1367781	14529570	AA	AB	AA	BB	Homozyg.	matIsodisomy
rs917440	15690278	BB	BB	BB	BB	Homozyg.	Probable Isodisomy
rs726395	15701240	BB	BB	BB	BB	Homozyg.	Probable Isodisomy

Additional file 5: Genotypes and inferred regions of isodisomy and heterodisomy

SNP ID	Position	Genotype Father	Genotype Mother	Genotype Sister	Genotype Patient	Homozygosity or Heterozygosity (Patient)	Isodisomy or Heterodisomy (Patient)
rs2030972	16025705	AA	AB	AA	BB	Homozyg.	matIsodisomy
rs1723804	16750469	AB	AB	AA	BB	Homozyg.	Probable Isodisomy
rs1375237	17532778	BB	BB	BB	BB	Homozyg.	Probable Isodisomy
rs957960	18843933	AA	AB	AA	AB	Heterozyg.	Probable Heterodisomy
rs714392	19577674	AA	AA	AA	AA	Homozyg.	Probable Heterodisomy
rs206198	20338283	AA	AA	AA	AA	Homozyg.	Probable Heterodisomy
rs1858941	20603004	BB	AA	AB	AA	Homozyg.	Probable Heterodisomy
rs2906667	21733521	AB	AB	AB	AB	Heterozyg.	Probable Heterodisomy
rs1859805	22150543	AB	AB	AB	AB	Heterozyg.	Probable Heterodisomy
rs1419756	22163506	AA	AB	AA	AB	Heterozyg.	Probable Heterodisomy
rs740295	22439813	AA	AA	AA	AA	Homozyg.	Probable Heterodisomy
rs858239	23252840	BB	AB	AB	AB	Heterozyg.	Probable Heterodisomy
rs798646	23586259	AB	AB	BB	AB	Heterozyg.	Probable Heterodisomy
rs1469000	23777130	AA	BB	AB	BB	Homozyg.	Probable Heterodisomy
rs39453	25100374	AB	AB	AB	AB	Heterozyg.	Probable Heterodisomy
rs766838	25996691	BB	AA	AB	AA	Homozyg.	Probable Heterodisomy
rs1404282	27551976	BB	BB	BB	BB	Homozyg.	Probable Heterodisomy
rs1860759	28364985	AA	AB	AB	AB	Heterozyg.	Probable Heterodisomy
rs7313	29036734	BB	AB	AB	AB	Heterozyg.	Probable Heterodisomy
rs1568658	29108083	AA	BB	AB	BB	Homozyg.	Probable Heterodisomy
rs245906	29166394	AB	AA	AB	AA	Homozyg.	Probable Heterodisomy
rs13834	29926987	AB	BB	AB	BB	Homozyg.	Probable Heterodisomy
rs255139	30719066	AA	AA	AA	AA	Homozyg.	Probable Heterodisomy
rs763486	31620009	AB	AB	AA	AB	Heterozyg.	Probable Heterodisomy
rs2049758	33041216	BB	AA	AB	AA	Homozyg.	Probable Heterodisomy
rs724291	33765662	AB	AA	AB	AA	Homozyg.	Probable Heterodisomy
rs731844	34116789	AB	AA	AB	AA	Homozyg.	Probable Heterodisomy
rs342990	35435570	AB	AB	AB	AB	Heterozyg.	Probable Heterodisomy
rs2041632	36432568	BB	AB	BB	AB	Heterozyg.	Probable Heterodisomy

Additional file 5: Genotypes and inferred regions of isodisomy and heterodisomy

SNP ID	Position	Genotype Father	Genotype Mother	Genotype Sister	Genotype Patient	Homozygosity or Heterozygosity (Patient)	Isodisomy or Heterodisomy (Patient)
rs2058675	36744857	BB	BB	BB	BB	Homozyg.	Probable Heterodisomy
rs918980	37568423	AA	AB	AA	AB	Heterozyg.	Probable Heterodisomy
rs2007664	37990365	BB	AA	AB	AA	Homozyg.	Probable Heterodisomy
rs722336	38471430	BB	BB	BB	BB	Homozyg.	Probable Heterodisomy
rs2043786	38497093	BB	BB	BB	BB	Homozyg.	Probable Heterodisomy
rs1949880	39062913	AB	AA	AB	AA	Homozyg.	Probable Heterodisomy
rs715283	39554757	BB	AB	BB	AB	Heterozyg.	Probable Heterodisomy
rs887447	40182550	BB	AB	BB	AB	Heterozyg.	Probable Heterodisomy
rs756328	40245787	AA	BB	AB	BB	Homozyg.	Probable Heterodisomy
rs722790	41000562	BB	BB	BB	BB	Homozyg.	Probable Heterodisomy
rs963779	41016114	AA	AA	AA	AA	Homozyg.	Probable Heterodisomy
rs1548630	41034306	BB	AB	BB	AB	Heterozyg.	Probable Heterodisomy
rs37262	41299419	AB	AA	AA	AA	Homozyg.	Probable Heterodisomy
rs273093	41389749	AB	BB	BB	BB	Homozyg.	Probable Heterodisomy
rs273196	41404546	AB	AA	AA	AA	Homozyg.	Probable Heterodisomy
rs2007475	41713082	AB	AB	AB	AB	Heterozyg.	Probable Heterodisomy
rs2024031	41857771	AA	AB	AA	AB	Heterozyg.	Probable Heterodisomy
rs7781467	41903433	AB	AB	BB	AB	Heterozyg.	Probable Heterodisomy
rs757911	41961362	AB	AA	AB	AA	Homozyg.	Probable Heterodisomy
rs846284	42036777	BB	BB	BB	BB	Homozyg.	Probable Heterodisomy
rs699491	42050798	BB	BB	BB	BB	Homozyg.	Probable Heterodisomy
rs846263	42058917	AA	AA	AA	AA	Homozyg.	Probable Heterodisomy
rs3823723	42105989	BB	AB	BB	AB	Heterozyg.	Probable Heterodisomy
rs1012033	43095049	AB	AB	BB	AB	Heterozyg.	Probable Heterodisomy
rs1029482	43221135	AA	AA	AA	AA	Homozyg.	Probable Heterodisomy
rs740094	44636223	BB	BB	BB	BB	Homozyg.	Probable Heterodisomy
rs1852210	45960781	AA	BB	AB	BB	Homozyg.	Probable Heterodisomy
rs2054789	46218057	BB	BB	BB	BB	Homozyg.	Probable Heterodisomy
rs1486155	46262242	AA	AA	AA	AA	Homozyg.	Probable Heterodisomy

Additional file 5: Genotypes and inferred regions of isodisomy and heterodisomy

SNP ID	Position	Genotype Father	Genotype Mother	Genotype Sister	Genotype Patient	Homozygosity or Heterozygosity (Patient)	Isodisomy or Heterodisomy (Patient)
rs723149	46543581	BB	AB	BB	AB	Heterozyg.	Probable Heterodisomy
rs2013523	47085660	AB	BB	BB	BB	Homozyg.	Probable Heterodisomy
rs921630	47761583	BB	AB	AB	AB	Heterozyg.	Probable Heterodisomy
rs1474283	47914388	AB	BB	AB	BB	Homozyg.	Probable Isodisomy
rs1529867	48785573	AA	AA	AA	AA	Homozyg.	Probable Isodisomy
rs1529590	49332707	AB	AA	AA	AA	Homozyg.	Probable Isodisomy
rs730652	50714171	AB	BB	BB	BB	Homozyg.	Probable Isodisomy
rs2043731	51137506	AB	AB	AB	AA	Homozyg.	Probable Isodisomy
rs616185	51804671	AB	AB	AB	BB	Homozyg.	Probable Isodisomy
rs169902	52620240	AB	AA	AB	AA	Homozyg.	Probable Isodisomy
rs2877132	53423097	BB	BB	BB	BB	Homozyg.	Probable Isodisomy
rs1524395	54165995	BB	AB	BB	BB	Homozyg.	Probable Isodisomy
rs2049410	54174050	BB	AB	BB	BB	Homozyg.	Probable Isodisomy
rs11238298	54186701	AA	AB	AA	AA	Homozyg.	Probable Isodisomy
rs12538489	55067330	BB	BB	BB	BB	Homozyg.	Probable Isodisomy
rs13222366	55967196	BB	AA	AB	AA	Homozyg.	Probable Isodisomy
rs1532084	56559246	BB	BB	BB	BB	Homozyg.	Probable Isodisomy
rs1532083	56560950	AA	AA	AA	AA	Homozyg.	Probable Isodisomy
rs6460119	63231970	BB	AB	BB	BB	Homozyg.	Probable Isodisomy
rs517258	64380618	AB	AA	AB	AA	Homozyg.	Probable Isodisomy
rs13536	65656625	AB	AB	BB	BB	Homozyg.	Probable Isodisomy
rs801193	65668047	AA	AB	AA	AA	Homozyg.	Probable Isodisomy
rs956523	66469861	AB	AB	AB	BB	Homozyg.	Probable Isodisomy
rs1874243	67466017	AA	AB	AA	AA	Homozyg.	Probable Isodisomy
rs1468588	68743592	BB	BB	BB	BB	Homozyg.	Probable Isodisomy
rs2533440	68747202	BB	BB	BB	BB	Homozyg.	Probable Isodisomy
rs3094901	69575886	BB	AB	BB	BB	Homozyg.	Probable Isodisomy
rs10266560	70403227	AB	AB	AB	AA	Homozyg.	Probable Isodisomy
rs678798	70827048	BB	BB	BB	BB	Homozyg.	Probable Isodisomy

Additional file 5: Genotypes and inferred regions of isodisomy and heterodisomy

SNP ID	Position	Genotype Father	Genotype Mother	Genotype Sister	Genotype Patient	Homozygosity or Heterozygosity (Patient)	Isodisomy or Heterodisomy (Patient)
rs2960920	71721683	AA	BB	AB	BB	Homozyg.	Probable Isodisomy
rs11764572	72816371	AB	AB	AB	AA	Homozyg.	Probable Isodisomy
rs7810996	73262551	BB	BB	BB	BB	Homozyg.	Probable Isodisomy
rs3135677	73293067	AA	AA	AA	AA	Homozyg.	Probable Isodisomy
rs13223937	73321316	BB	AB	BB	BB	Homozyg.	Probable Isodisomy
rs2527367	73737074	AA	AB	AB	BB	Homozyg.	matIsodisomy
rs757364	75125346	AB	AA	AB	AA	Homozyg.	Probable Isodisomy
rs1859293	75210915	AB	AA	AB	AA	Homozyg.	Probable Isodisomy
rs917424	75822468	AA	AB	AB	BB	Homozyg.	matIsodisomy
rs10224066	75842645	BB	BB	BB	BB	Homozyg.	Probable Isodisomy
rs740158	76893772	AA	AB	AB	BB	Homozyg.	matIsodisomy
rs798332	77746864	AA	AA	AA	AA	Homozyg.	Probable Isodisomy
rs714438	77820241	AA	AA	AA	AA	Homozyg.	Probable Isodisomy
rs1799003	78403616	AA	AB	AA	AA	Homozyg.	Probable Isodisomy
rs1034699	79544858	AA	AA	AA	AA	Homozyg.	Probable Isodisomy
rs1468242	79636560	AA	AA	AA	AA	Homozyg.	Probable Isodisomy
rs2030711	80057878	AA	AA	AA	AA	Homozyg.	Probable Isodisomy
rs1029847	81431156	AB	AB	BB	BB	Homozyg.	Probable Isodisomy
rs258651	81559449	AA	AA	AA	AA	Homozyg.	Probable Isodisomy
rs28156	82484146	AA	AA	AA	AA	Homozyg.	Probable Isodisomy
rs42002	83099021	AA	BB	AB	BB	Homozyg.	Probable Isodisomy
rs917089	83568385	AB	AB	AA	AA	Homozyg.	Probable Isodisomy
rs473880	83747016	BB	AB	BB	BB	Homozyg.	Probable Isodisomy
rs612774	83820122	AA	AA	AA	AA	Homozyg.	Probable Isodisomy
rs764077	84602330	AA	AA	AA	AA	Homozyg.	Probable Isodisomy
rs1024516	86119134	AB	AB	AA	AA	Homozyg.	Probable Isodisomy
rs4148738	87000985	AA	BB	AB	BB	Homozyg.	Probable Isodisomy
rs1202169	87033786	BB	BB	BB	BB	Homozyg.	Probable Isodisomy
rs1637503	87430579	AB	AA	AA	AA	Homozyg.	Probable Isodisomy

Additional file 5: Genotypes and inferred regions of isodisomy and heterodisomy

SNP ID	Position	Genotype Father	Genotype Mother	Genotype Sister	Genotype Patient	Homozygosity or Heterozygosity (Patient)	Isodisomy or Heterodisomy (Patient)
rs1688886	87441679	AB	BB	BB	BB	Homozyg.	Probable Isodisomy
rs1637489	87451497	AB	BB	BB	BB	Homozyg.	Probable Isodisomy
rs1468121	88185805	BB	BB	BB	BB	Homozyg.	Probable Isodisomy
rs717474	88629233	AB	BB	BB	BB	Homozyg.	Probable Isodisomy
rs1023564	88679650	AA	AB	AB	BB	Homozyg.	matIsodisomy
rs194506	89680566	AA	AA	AA	AA	Homozyg.	Probable Isodisomy
rs194518	89691085	BB	BB	BB	BB	Homozyg.	Probable Isodisomy
rs42611	89787996	AB	BB	AB	BB	Homozyg.	Probable Isodisomy
rs758706	90332855	AB	AB	AB	AA	Homozyg.	Probable Isodisomy
rs722263	92724748	BB	AA	AB	AA	Homozyg.	Probable Isodisomy
rs1326152	94273042	BB	BB	BB	BB	Homozyg.	Probable Isodisomy
rs854731	94471624	AA	AA	AA	AA	Homozyg.	Probable Isodisomy
rs1859121	94814903	BB	AB	BB	BB	Homozyg.	Probable Isodisomy
rs1053275	94839491	AA	AB	AA	AA	Homozyg.	Probable Isodisomy
rs1917486	96079539	BB	AA	AB	AA	Homozyg.	Probable Isodisomy
rs1229540	97261330	AA	AB	AB	BB	Homozyg.	matIsodisomy
rs1047035	98282223	BB	AB	BB	BB	Homozyg.	Probable Isodisomy
rs219826	98485075	AA	AB	AB	BB	Homozyg.	matIsodisomy
rs219798	98488035	BB	AB	AB	AA	Homozyg.	matIsodisomy
rs4727439	99348170	AB	BB	AB	BB	Homozyg.	Probable Isodisomy
rs1617640	100155234	AA	AA	AA	AA	Homozyg.	Probable Isodisomy
rs11178	100567804	BB	AA	AB	AA	Homozyg.	Probable Isodisomy
rs201492	101540858	AB	AB	AA	BB	Homozyg.	Probable Isodisomy
rs727708	103118180	AB	AB	AA	BB	Homozyg.	Probable Isodisomy
rs39400	103281118	BB	BB	BB	BB	Homozyg.	Probable Isodisomy
rs1010340	104617931	AA	AA	AA	AA	Homozyg.	Probable Isodisomy
rs234	105348371	AB	AB	AB	AA	Homozyg.	Probable Isodisomy
rs41261	105399068	AB	BB	BB	BB	Homozyg.	Probable Isodisomy
rs176481	105515161	BB	AB	BB	AA	Homozyg.	matIsodisomy

Additional file 5: Genotypes and inferred regions of isodisomy and heterodisomy

SNP ID	Position	Genotype Father	Genotype Mother	Genotype Sister	Genotype Patient	Homozygosity or Heterozygosity (Patient)	Isodisomy or Heterodisomy (Patient)
rs887882	105622519	AB	AB	AB	AA	Homozyg.	Probable Isodisomy
rs1024761	105875514	BB	AB	AB	BB	Homozyg.	Probable Isodisomy
rs1476878	106485904	AA	AB	AB	AA	Homozyg.	Probable Isodisomy
rs257376	106587233	AA	AB	AB	AA	Homozyg.	Probable Isodisomy
rs2028030	107147241	AB	BB	BB	BB	Homozyg.	Probable Isodisomy
rs441534	107616772	AB	AA	AA	AA	Homozyg.	Probable Isodisomy
rs10234165	108674847	AA	AA	AA	AA	Homozyg.	Probable Isodisomy
rs1013920	109655311	AB	AB	AA	BB	Homozyg.	Probable Isodisomy
rs719530	109678201	AB	BB	AB	BB	Homozyg.	Probable Isodisomy
rs214459	110637675	AB	BB	BB	BB	Homozyg.	Probable Isodisomy
rs214468	110649119	AB	BB	BB	BB	Homozyg.	Probable Isodisomy
rs37742	110700856	AB	BB	BB	BB	Homozyg.	Probable Isodisomy
rs1476517	111285062	AB	AB	AA	BB	Homozyg.	Probable Isodisomy
rs7817	111902894	BB	BB	BB	BB	Homozyg.	Probable Isodisomy
rs2966478	112048462	BB	BB	BB	BB	Homozyg.	Probable Isodisomy
rs2940339	112973745	AB	BB	AB	BB	Homozyg.	Probable Isodisomy
rs1123065	114452015	BB	BB	BB	BB	Homozyg.	Probable Isodisomy
rs2040587	114462759	BB	AB	BB	AA	Homozyg.	matIsodisomy
rs2056865	116007768	AB	BB	BB	BB	Homozyg.	Probable Isodisomy
rs41736	116223004	AB	BB	AB	BB	Homozyg.	Probable Isodisomy
rs885993	116916640	AA	AB	AA	BB	Homozyg.	matIsodisomy
rs213950	116986769	BB	AB	BB	AA	Homozyg.	matIsodisomy
rs38831	117507112	AB	BB	BB	BB	Homozyg.	Probable Isodisomy
rs846427	119280806	BB	BB	BB	BB	Homozyg.	Probable Isodisomy
rs868053	120567521	BB	BB	BB	BB	Homozyg.	Probable Isodisomy
rs12217	121501889	AB	AA	AA	AA	Homozyg.	Probable Isodisomy
rs1320393	122558943	AB	AB	AB	AA	Homozyg.	Probable Isodisomy
rs1880180	122626098	AB	AB	AB	AA	Homozyg.	Probable Isodisomy
rs1121030	123495485	AA	AB	AB	AA	Homozyg.	Probable Isodisomy

Additional file 5: Genotypes and inferred regions of isodisomy and heterodisomy

SNP ID	Position	Genotype Father	Genotype Mother	Genotype Sister	Genotype Patient	Homozygosity or Heterozygosity (Patient)	Isodisomy or Heterodisomy (Patient)
rs1860487	123502372	AA	AA	AA	AA	Homozyg.	Probable Isodisomy
rs648619	124591104	AB	AB	AB	AA	Homozyg.	Probable Isodisomy
rs1833083	125007189	AA	AA	AA	AA	Homozyg.	Probable Isodisomy
rs996867	125034557	AA	AB	AA	BB	Homozyg.	matIsodisomy
rs1419607	125178881	BB	BB	BB	BB	Homozyg.	Probable Isodisomy
rs719319	125499973	AB	AA	AA	AA	Homozyg.	Probable Isodisomy
rs766240	125922975	AB	BB	BB	BB	Homozyg.	Probable Isodisomy
rs1419438	126416116	AA	BB	AB	BB	Homozyg.	Probable Isodisomy
rs1419437	126447341	AA	BB	AB	BB	Homozyg.	Probable Isodisomy
rs322812	127532023	AA	AA	AA	AA	Homozyg.	Probable Isodisomy
rs587499	128864767	BB	BB	BB	BB	Homozyg.	Probable Isodisomy
rs1464890	129451548	BB	BB	BB	BB	Homozyg.	Probable Isodisomy
rs2030974	129693119	AA	AA	AA	AA	Homozyg.	Probable Isodisomy
rs729332	129704366	AA	AB	AA	BB	Homozyg.	matIsodisomy
rs1809627	129758424	BB	BB	BB	BB	Homozyg.	Probable Isodisomy
rs1990790	129820866	AB	AA	AA	AA	Homozyg.	Probable Isodisomy
rs969827	130291151	AA	BB	AB	BB	Homozyg.	Probable Isodisomy
rs15956	130831458	AB	AB	AB	AA	Homozyg.	Probable Isodisomy
rs4728251	131478876	AA	AB	AA	BB	Homozyg.	matIsodisomy
rs10223934	132042784	AB	AB	AA	BB	Homozyg.	Probable Isodisomy
rs1468974	132230234	AA	AB	AB	AA	Homozyg.	Probable Isodisomy
rs889826	133246411	AB	AA	AB	AA	Homozyg.	Probable Isodisomy
rs1646656	133681457	BB	BB	BB	BB	Homozyg.	Probable Isodisomy
rs1962522	134436889	AB	BB	AB	BB	Homozyg.	Probable Isodisomy
rs1559534	134692033	BB	AB	AB	BB	Homozyg.	Probable Isodisomy
rs1863005	134704002	AA	AB	AA	BB	Homozyg.	matIsodisomy
rs1582634	134817061	AA	AB	AA	BB	Homozyg.	matIsodisomy
rs959520	135922473	AA	AA	AA	AA	Homozyg.	Probable Isodisomy
rs10488598	136238383	AA	AB	AB	AA	Homozyg.	Probable Isodisomy

Additional file 5: Genotypes and inferred regions of isodisomy and heterodisomy

SNP ID	Position	Genotype Father	Genotype Mother	Genotype Sister	Genotype Patient	Homozygosity or Heterozygosity (Patient)	Isodisomy or Heterodisomy (Patient)
rs748260	137136608	AA	AA	AA	AA	Homozyg.	Probable Isodisomy
rs273954	137254086	AB	BB	AB	BB	Homozyg.	Probable Isodisomy
rs1424376	137338529	BB	BB	BB	BB	Homozyg.	Probable Isodisomy
rs1371463	137933620	AB	AA	AA	AA	Homozyg.	Probable Isodisomy
rs1015343	138003727	AB	BB	AB	BB	Homozyg.	Probable Isodisomy
rs3778931	138753030	AB	BB	AB	BB	Homozyg.	Probable Isodisomy
rs1464798	138982540	BB	BB	BB	BB	Homozyg.	Probable Isodisomy
rs880290	139567990	BB	BB	BB	BB	Homozyg.	Probable Isodisomy
rs1476640	141058779	AB	AB	AA	AB	Heterozyg.	Probable Heterodisomy
rs768055	141059520	BB	AB	BB	AB	Heterozyg.	Probable Heterodisomy
rs1859646	141142073	AA	AB	AA	AB	Heterozyg.	Probable Heterodisomy
rs940864	142749433	AA	AB	AA	AB	Heterozyg.	Probable Heterodisomy
rs727714	143729925	AA	AA	AA	AA	Homozyg.	Probable Heterodisomy
rs969356	143804256	BB	BB	BB	BB	Homozyg.	Probable Heterodisomy
rs2056553	144893341	BB	AB	AB	AB	Heterozyg.	Probable Heterodisomy
rs850545	145027074	AA	BB	AB	BB	Homozyg.	Probable Heterodisomy
rs1860482	145049320	BB	BB	BB	BB	Homozyg.	Probable Heterodisomy
rs802200	145736404	BB	AA	AB	AA	Homozyg.	Probable Heterodisomy
rs700273	145807313	BB	AA	AB	AA	Homozyg.	Probable Heterodisomy
rs1524341	146337622	AA	AA	AA	AA	Homozyg.	Probable Heterodisomy
rs1024676	146346794	AB	AB	AB	AB	Heterozyg.	Probable Heterodisomy
rs10240438	146513587	AB	AA	AA	AA	Homozyg.	Probable Heterodisomy
rs1496542	146534867	AB	BB	BB	BB	Homozyg.	Probable Heterodisomy
rs4431523	147228099	AB	AA	AB	AA	Homozyg.	Probable Heterodisomy
rs963314	147493565	AA	AA	AA	AA	Homozyg.	Probable Heterodisomy
rs243491	148077106	BB	AB	BB	AB	Heterozyg.	Probable Heterodisomy
rs740136	149052143	AB	BB	AB	BB	Homozyg.	Probable Heterodisomy
rs757723	149078087	AA	AB	AA	AB	Heterozyg.	Probable Heterodisomy
rs1104881	149772742	BB	AB	AB	AB	Heterozyg.	Probable Heterodisomy

Additional file 5: Genotypes and inferred regions of isodisomy and heterodisomy

SNP ID	Position	Genotype Father	Genotype Mother	Genotype Sister	Genotype Patient	Homozygosity or Heterozygosity (Patient)	Isodisomy or Heterodisomy (Patient)
rs1547958	150271218	BB	BB	BB	BB	Homozyg.	Probable Heterodisomy
rs875588	150799763	AB	BB	AB	BB	Homozyg.	Probable Heterodisomy
rs2536077	151040039	BB	AB	BB	AB	Heterozyg.	Probable Heterodisomy
rs10235893	151809888	BB	AA	AB	AA	Homozyg.	Probable Heterodisomy
rs880310	151832710	BB	BB	BB	BB	Homozyg.	Probable Heterodisomy
rs6951978	152518421	AB	BB	BB	BB	Homozyg.	Probable Heterodisomy
rs1735093	152628820	AB	BB	AB	BB	Homozyg.	Probable Heterodisomy
rs38993	153207190	AB	BB	AB	BB	Homozyg.	Probable Heterodisomy
rs1073088	153725713	BB	AB	AB	AB	Heterozyg.	Probable Heterodisomy
rs7800754	154009264	BB	AB	AB	AB	Heterozyg.	Probable Heterodisomy
rs10266693	154080711	AA	AA	AA	AA	Homozyg.	Probable Heterodisomy
rs306278	154419731	AA	AB	AB	AB	Heterozyg.	Probable Heterodisomy
rs1657290	154589637	AB	BB	BB	BB	Homozyg.	Probable Heterodisomy
rs10279206	154906561	AA	AA	AA	AA	Homozyg.	Probable Heterodisomy
rs6953751	155070191	BB	AB	AB	AB	Heterozyg.	Probable Heterodisomy
rs1920454	155196211	BB	AA	AB	AA	Homozyg.	Probable Heterodisomy
rs1343750	155214887	AA	BB	AB	BB	Homozyg.	Probable Heterodisomy
rs1531381	155383699	BB	BB	BB	BB	Homozyg.	Probable Heterodisomy
rs10949794	155503432	BB	BB	BB	BB	Homozyg.	Probable Heterodisomy
rs1389240	155745559	AB	AB	AB	AB	Heterozyg.	Probable Heterodisomy
rs1182378	156741083	AB	AB	AB	AB	Heterozyg.	Probable Heterodisomy
rs1182414	156751124	AB	AB	AB	AB	Heterozyg.	Probable Heterodisomy
rs917740	156945610	AB	AB	AB	AB	Heterozyg.	Probable Heterodisomy
rs7455229	157525204	AA	AB	AB	AB	Heterozyg.	Probable Heterodisomy
rs6953748	157813945	AA	AA	AA	AA	Homozyg.	Probable Heterodisomy
rs10772	158116888	BB	AB	BB	AB	Heterozyg.	Probable Heterodisomy
rs455030	158710965	AA	AB	AA	AB	Heterozyg.	Probable Heterodisomy

Additional file 5: Genotypes and inferred regions of isodisomy and heterodisomy

SNP ID	Position	Genotype Father	Genotype Mother	Genotype Sister	Genotype Patient	Homozygosity or Heterozygosity (Patient)	Isodisomy or Heterodisomy (Patient)

Cells in red: Genetic discordance in inheritance
Cells in dark green: Unambiguous maternal uniparental disomy
Cells in light green: Probable maternal uniparental disomy
Cells in blue: Probable uniparental heterodisomy
Cells in yellow: Genotype shared between the patient and his sister
